# Potato consumption and the risk of overall and cause specific mortality in the NIH-AARP study

**DOI:** 10.1371/journal.pone.0216348

**Published:** 2019-05-07

**Authors:** Maryam Hashemian, Gwen Murphy, Arash Etemadi, Linda M. Liao, Sanford M. Dawsey, Reza Malekzadeh, Christian C. Abnet

**Affiliations:** 1 Metabolic Epidemiology Branch, Division of Cancer Epidemiology and Genetics, National Cancer Institute, National Institutes of Health, Bethesda, United States of America; 2 Digestive Oncology Research Center, Digestive Diseases Research Institute, Tehran University of Medical Sciences, Tehran, Iran; 3 Digestive Disease Research Center, Digestive Diseases Research Institute, Tehran University of Medical Sciences, Tehran, Iran; Emory University, UNITED STATES

## Abstract

**Background:**

Potato consumption has been hypothesized to be associated with higher risk of hypertension, diabetes, and colorectal cancer.

**Objective:**

The aim of this study was to examine the association between potato consumption and the risk of overall and cause specific mortality in the large prospective National Institutes of Health–AARP (NIH-AARP) Study.

**Design:**

The NIH-AARP study recruited 566,407 persons, aged 50–72 years in 1995–1996. We excluded subjects that reported a history of chronic disease at baseline. Potato consumption data from a validated food frequency questionnaire completed at baseline was used in Cox proportional hazard models to estimate hazard ratios (HR) and 95% confidence intervals (95% CI) for overall and cause specific mortality. Final models were adjusted for potential risk factors for mortality.

**Results:**

Among 410,701 participants included in this analysis, 76,921 persons died during the 15.6 years of follow-up. Eating baked, boiled, or mashed potatoes, French fries or potato salad seven or more times per week was associated with higher risk of overall mortality, in models adjusted only for age and sex (HR _C4 vs C1_ = 1.17, 95%CI = 1.13, 1.21). These results were attenuated in fully adjusted models (HR _C4 vs C1_ = 1.02, 95%CI = 0.97, 1.06). Potato consumption was not associated with risk of mortality caused by cancer (HR _C4 vs C1_ = 1.04, 95%CI = 0.97, 1.11), heart disease (HR _C4 vs C1_ = 1.00, 95%CI = 0.93, 1.09), respiratory disease (HR _C4 vs C1_ = 1.16, 95%CI = 0.99, 1.37), or diabetes (HR _C4 vs C1_ = 0.91, 95%CI = 0.71, 1.19). We tested for an association with different preparation methods and found limited evidence for differences by preparation method. The only statistically significant association was that for French fry consumption with cancer-related mortality (HR _C4 vs C1_ = 1.27, 95%CI = 1.02, 1.59), a finding for which uncontrolled confounding could not be ruled out.

**Conclusion:**

We find little evidence that potato consumption is associated with all-cause or cause-specific mortality.

## Introduction

Potatoes are the most common non-cereal staple food consumed worldwide [1]. US per capita potato usage exceeds 100 pounds per year, with an increasing fraction sold processed rather than fresh [2]. Potatoes have a high glycemic index and high glycemic load and may be perceived to be unhealthy, which may be partly due to the preparation methods, such as deep frying, or because of the condiments added during serving, such as butter, cheese, or sour cream, which may add a substantial amount of fat and energy. However, they are a good source of potassium and polyphenols [3] and can be a concentrated source of other required nutrients. For example, one serving of white potatoes provides about one-third of the Recommended Dietary Allowance (RDA) for vitamin C, but only 147 kcal of energy. The widespread popularity of potatoes and their mixed nutritional reputation have made potatoes the focus on an ongoing debate within the nutrition community [4]. Potatoes are included as total vegetables in Healthy Eating Index but are not included in most of dietary indices [5]. Because randomized trials are unlikely to be used to resolve this debate, more data from large, long-term observational studies may be the best way to add to our understanding of the role of potatoes in a healthy diet.

Three prospective studies in the US (the Nurses’ Health Studies 1 and 2, and the Health Professionals’ Follow-up Study) reported that higher consumption of potatoes is associated with an higher risk of diabetes mellitus and higher risk of hypertension, independent of body mass index (BMI) and other risk factors [6, 7]. However, two prospective studies in Sweden reported that potato consumption is not associated with risk of incident cardiovascular disease [3], and a study assessed two different Spanish prospective studies reported that potato consumption is not associated with the risk of hypertension [8]. A Norwegian study reported that high potato consumption is associated with higher risk of colorectal cancer [9]. Another recent study reported that consumption of French fries is associated with higher risk of mortality, but total potato consumption was not associated with mortality. However, the results of this study were based on only 236 deaths [10]. Therefore, the aim of our study was to evaluate the associations of potato consumption with overall and cause-specific mortality in the NIH-AARP Diet and Health Study, which has accrued 76,921 deaths among healthy participants at baseline during 5,942,912 person-years of follow-up between 1995–2011.

## Materials and methods

### Study design

The NIH-AARP Diet and Health Study was launched in 1995 and enrolled AARP members living in California, Florida, Louisiana, New Jersey, North Carolina, and Pennsylvania and two metropolitan areas (Atlanta, Georgia, and Detroit, Michigan). A demographic questionnaire and a food frequency questionnaire were mailed to 3.5 million AARP members. Subjects were 50 to 71 years old. In total, 617,119 baseline questionnaires were returned (an 18% response rate). The details were described previously [11]. The data underlying the results presented in the study are available upon submitting a proposal to be approved by the NIH-AARP Steering Committee at https://www.nihaarpstars.com.

#### Participants

The NIH-AARP baseline cohort encompassed 566,398 subjects with satisfactorily completed questionnaires. In this analysis, proxy respondents (n = 15,760); participants outside two interquartile ranges of the 25th and 75th percentile interval of the energy intake (n = 4809); subjects with any registry confirmed cancer diagnosis before study entry (n = 8,828); and self-reported history of cancer (n = 42,165), heart disease (n = 68,408), stroke (n = 6,360), and emphysema (n = 9,367) were excluded. In total, 410,701 subjects (233,739 men and 176,962 women) aged 50–71 years were included for the present analysis **(S1 Fig).**

The NIH-AARP Diet and Health Study is conducted under the auspices of the Special Studies Institutional Review Board of the United States National Cancer Institute, and all subjects provided written informed consent.

### Cohort follow-up

Participants were followed from baseline for each individual (1995–1996) until the date of death, loss to follow-up, withdrawal from the study, or December 31, 2011, whichever came first.

### Exposure assessment

Demographic and anthropometric information were collected at baseline using a self-administered questionnaire. The 124-item food frequency questionnaire [12] was also mailed at baseline, and the participants were asked to report their consumption in the past 12 months. The validity and reproducibility of the food frequency questionnaire was previously reported, and for the 26 nutrient constituents examined, estimated correlations with true intake ranged from 0.36 to 0.76 [12]. Potato consumption was asked using five questions: “baked, boiled, or mashed potatoes”; “potato salad”; “French fries or home fries”; “sweet potatoes”; and “chips”. Total potato consumption was computed by summing the responses to the first three questions. For this study, we did not include sweet potatoes, because they have a different nutrient content. We also did not include chips, because the question did not differentiate between potato, corn, or other chips.

The frequency of potato consumption consisted of 10 categories from never to “2 or more times per day”. Each item in the food frequency questionnaire was linked to the 1994–1996 US Department of Agriculture's Continuing Survey of Food Intakes by Individuals to compute energy and nutrient intake [13].

### Outcome measures

Vital status and cause-specific mortality were ascertained using probabilistic matching to the National Death Index Plus. The International Classification of Diseases, 10th revision (ICD-10) was used to classify the underlying cause of death (from death certificates). We studied death from cancer (ICD-10 codes C00-C44, C45.0, C45.1, C45.7, C45.9, C48-C97, and D12-D48); cardiovascular disease, including heart diseases (ICD-10 codes I00-I09, I10-I13, I20-I51, and I70-I78) and stroke or cerebrovascular diseases (ICD-10 codes I60-I69); respiratory diseases (ICD-10 codes J10-J18 and J40-J47); and diabetes mellitus (ICD-10 codes E10-E14).

### Statistical analysis

Descriptive values of variables were expressed as means and percentages. Cox proportional hazards models were used to estimate hazard ratios (HRs) and 95% confidence intervals (95% CIs). Time since entry to the study was used as the underlying time metric. Using age at entry did not change the results significantly. The proportional hazards assumption was verified using the Schoenfeld residuals test.

Ten categories of potato consumption were merged into 4 categories: less than 1 time per week, 1–2 times per week, 3–6 times per week, 7 or more times per week. In a secondary analysis, we merged the top two categories to render more evenly distributed categories for potato consumption. The lowest category was used as a reference category throughout. To test for linear trend across categories of intake, the median value of each category was used as a continuous variable in the regression models. For continuous analyses, we estimated the HRs associated with a one serving/day increase in potato intake.

Multivariable models were adjusted for suspected risk factors for mortality including age; sex; body mass index (BMI); ethnic group; education; drinking alcohol; smoking status, intensity and duration; self-reported health status; self-report of diabetes (yes vs. no); level of physical activity (never, rarely, 1–3 times/month, 1–2 times/week, 3–4 times/week, >5 times/week); use of any vitamin supplement (yes vs. no); total energy intake (quintile); consumption of red meat (quintile), white meat (quintile), whole grains (quintile), fruits (quintile) and vegetables (quintile). We also tested the inclusion of the healthy eating index 2010 in the model instead of individual foods (red meat, white meat, whole grains, fruits and vegetables) and found no substantive differences in the results from the primary models we present.

In additional analyses, we mutually adjusted for each type of potato preparation (including French fries in the model for baked potato and potato salad and vice versa). Also, we converted the potato intake to grams per day and adjusted the intake for energy using the nutrient density method. We did stratified analyses by potential effect modifiers (age, smoking status, BMI, self-reported health status, red meat intake, vitamin supplement use), and tested the interaction between potato consumption and effect modifiers using the Likelihood Ratio test. Finally, we conducted a sensitivity analysis in which we excluded participants with less than two years of follow-up, to evaluate potential reverse causality.

Statistical analyses were performed with STATA software version 14 (STATA Corp, College Station, TX, USA). P values less than 0.05 or 95% confidence intervals that excluded 1.0 were considered statistically significant.

## Results

During the 15.6 years of follow up, 76,921 participants were reported to have died (48,830 men and 28,091 women). The baseline characteristics of these participants by potato consumption categories are provided in **Table 1**. Overall, potato consumption was associated with a host of other variables, many of which may confer higher risk of death. Specifically, individuals with higher potato consumption were more likely to be current smokers, to be non-Hispanic whites, to drink more alcohol, to have a history of diabetes, and to report poor or fair health. They were also less likely to have attended college or to use vitamin-mineral supplements. Potato consumption was associated with higher energy intake; higher red meat, white meat, whole grain, fruit and vegetable intake; and a higher BMI. The baseline characteristics of participants by different types of potato preparation categories are provided in **S1 Table**.

**Table 1:** Demographic and dietary characteristics of participants by potato consumption in the NIH-AARP diet and health study^1^.

	Men				Women			
	< 1 time per week	1–2 times per week	3–6 times per week	≥7 times per week	< 1 time per week	1–2 times per week	3–6 times per week	≥7 times per week
**Participants, n**	66,130	85,753	72,325	9,531	62,220	61,234	48,665	4,843
**Total potato intake, gram per day ^2^**	13.5±7.6	37±14.6	85.9±38	161.6±75.2	10.6±6.2	30.7±11.4	74.5±31.1	142.3±68.3
**Age, years ^2^**	62.2±5.3	62.1±5.4	62.6±5.3	62.3±5.4	61.8±5.4	61.8±5.4	62.3±5.3	62.3±5.4
**BMI ^2^, kg/m^2^**	27.2±4.3	27.3±4.2	27.3±4.3	27.6±4.8	26.7±6.1	26.9±5.9	27.0±6.2	27.4±6.7
**Smoking (%)**								
** Never**	25,362 (30)	34,044 (30.7)	28,426 (29.6)	3,699 (29.6)	31,900 (43.3)	33,431 (45.6)	27,689 (46.5)	2,927 (48.1)
** Former <20 cigarettes per day**	26368 (31.2)	33,036 (29.7)	28,101 (29.2)	3,352 (26.9)	21,264 (28.8)	20,156 (27.5)	15,635 (26.3)	1,449 (23.8)
** Former ≥20 cigarettes per day**	24,062 (28.5)	32,660 (29.4)	28,696 (29.9)	3,755 (30.1)	9,538 (12.9)	9,307 (12.7)	7,435 (12.5)	713 (11.7)
** Current <20 cigarettes per day**	5,330 (6.3)	6,562 (5.9)	5,913 (6.1)	901 (7.2)	8,196 (11.1)	7,669 (10.4)	6,094 (10.2)	709 (11.6)
** Current ≥20 cigarettes per day**	3,405 (4.0)	4,777 (4.3)	4,941 (5.1)	770 (6.2)	2,802 (3.8)	2,815 (3.8)	2,660 (4.5)	287 (4.7)
**Alcohol, grams per day ^2^**	15.8±40.1	16.6±37.5	17.5±38.6	17.5±40.7	5.5±15.8	5.9±15.7	6.2±17.1	5.9±17.9
**Race (%)**								
** Non-Hispanic white**	76,655 (88.1)	108,347 (94.9)	95,745 (96.9)	12,432 (96.6)	64,161 (88.3)	69,602 (93.0)	57,991 (95.4	5,895 (94.4)
** Non-Hispanic Black**	4,416 (5.1)	2,536 (2.2)	1,412 (1.4)	179 (1.4)	6,762 (9.0)	3,246 (4.3)	1,741 (2.9)	221 (3.5)
** Others**	5,991 (6.9)	3,262 (2.9)	1,622 (1.6)	258 (2.0)	4,259 (5.7)	2,011 (2.7)	1,052 (1.7)	129 (2.1)
**Education, College and post-graduate (%)**	40,733 (47.5)	53,765 (47.8)	41,946 (43.1)	4,709 (37.3)	24,181 (32.5)	23,850 (32.4)	16,630 (27.9)	1,363 (22.4)
**Physical activity, ≥ 5 times per week (%)**	18,749 (21.5)	24,102 (21.1)	21,387 (21.6)	2,907 (22.5)	12,530 (16.6)	11,804 (15.7)	10,078 (16.6)	1,063 (17.0)
**Self-reported history of diabetes, yes (%)**	9,111 (10.3)	11,170 (9.7)	10,577 (10.6)	1,682 (12.9)	5,612 (7.3)	5 504 (7.3)	4,904 (8.0)	619 (9.8)
**Self-reported poor or fair health (%)**	11,045 (12.7)	13,473 (11.8)	13,031 (13.2)	2,009 (15.6)	10,240 (13.6)	9,674 (13.0)	8,612 (14.2)	1,183 (19.1)
**Use of any vitamin mineral supplement (%)**	47,111 (53.2)	60,341 (52.3)	50,598 (50.7)	6,204 ((47.6)	46,837 (61)	46,312 (61.1)	36,581 (59.5)	3,496 (55.1)
**Calories, kcal/day ^2^**	1646±733	1957±751	2290±839	2886±1.30	1314±571	1560±584	1825±667	2309±851
**Red meat intake, grams per day ^2^**	54.5±47.6	75.8±55.0	95.0±66.8	130.5±90.4	33.9±31.7	48.0±37.1	59.2±59.2	80.9±64.1
**White meat intake, grams per day ^2^**	51.0±49.3	62.2±50.5	70.2±56.5	82.2±70.9	46.9±48.4	56.1±46.3	63.8±53.2	73.5±67.7
**Whole grain intake, grams per day ^2^**	20.3±29.0	23.4±29.5	26.8±33.3	31.3±40.0	17.7±25.2	20.8±25.9	23.7±28.6	27.8±35.6
**Fruit intake, grams per day ^2^**	360.4±354.0	370.3±333.0	387.0±343.6	424.7±404.3	340.11±328.7	354.2±304.1	383.1±324.7	441.4±405.2
**Vegetable intake, grams per day ^2^^,^^3^**	209.2±178.1	246.7±172.0	286.2±193.1	359.0±268.6	209.6±177.9	248.3±171.1	292.7±202.8	362.8±266.8

^1^ All risk factors were associated with potato consumption with p <0.001

^2^ Mean ±SD

^3^ Excluding potatoes

Table 2 presents the age- and sex-adjusted and fully adjusted HRs for overall and cause-specific mortality for increasing categories of potato consumption. The age- and sex-adjusted models suggested that participants who ate potatoes seven or more times per week had higher risk of overall and cause-specific mortality in comparison with those who ate potatoes less than one time per week, with highly significant p-values for trend across categories. After adjusting for multiple risk factors, the estimated associations were attenuated to the null and were no longer statistically significant. The apparent overall association with mortality was reduced to non-significance (HR _C4 vs C1_ = 1.02, 95%CI = 0.97, 1.06). Merging the top two categories of potato consumption did not change the results meaningfully (HR _C3 vs C1_ = 0.97, 95%CI = 0.95, 1.00). when we analyzed potato intake as a continuous variable, the estimated fully-adjusted associations were close to null and not statistically significant (Table 2).

**Table 2:** Age and sex adjusted and fully adjusted hazard ratios for overall and cause specific mortality, for categories of total potato consumption (n = 410,701).

Cause of death	Categories of potato consumption					
	< 1 time per week	1–2 times per week	3–6 times per week	≥7 times per week	P trend	1 serving increase/ day
**All causes of death**						
Death, n/ person-year	25,220/1,834,840	27,980/2,100,434	25,511/1,737,904	3,502/207,774		
Age- and sex- adjusted HR	1	0.95 (0.94, 0.97)	1.01 (1.00, 1.03)	1.17 (1.13, 1.21)	<0.001	1.07 (1.06, 1.08)
Fully adjusted HR ^1^	1	0.98 (0.96, 1.00)	1.00 (0.98, 1.02)	1.02 (0.97, 1.06)	0.02	1.00 (0.98, 1.01)
**Cancer**						
Death, n/ person-year	9,523/1,834,840	10,751/2,100,434	9,639/1,737,904	1,276/207,774		
Age- and sex- adjusted HR	1	0.97 (0.94, 1.00)	1.02 (0.99, 1.05)	1.15 (1.09, 1.22)	<0.001	1.07 (1.06, 1.09)
Fully adjusted HR^1^	1	0.95 (0.95, 1.01)	0.99 (0.96, 1.02)	1.04 (0.97, 1.11)	0.21	1.01 (0.99, 1.04)
**Heart disease**						
Death, n/ person-year	6,903/1,834,840	7,570/2,100,434	7,055/1,737,904	978/207,774		
Age- and sex- adjusted HR	1	0.94 (0.91, 0.97)	1.01 (0.98, 1.05)	1.17 (1.10, 1.26)	<0.001	1.07 (1.05, 1.09)
Fully adjusted HR^1^	1	0.98 (0.94, 1.01)	1.02 (0.98, 1.06)	1.00 (0.93, 1.09)	0.08	1.01 (0.98, 1.03)
**Respiratory disease**						
Death, n/ person-year	2,011/1,834,840	2,322/2,100,434	2,294/1,737,904	337/207,774		
Age- and sex- adjusted HR	1	1.01 (0.95, 1.08)	1.16 (1.09, 1.23)	1.40 (1.22, 1.62)	<0.001	1.12 (1.07, 1.17)
Fully adjusted HR^1^	1	1.02 (0.94, 1.11)	1.01 (0.93, 1.10)	1.16 (0.99, 1.37)	0.21	0.99 (0.94, 1.05)
**Diabetes**						
Death, n/ person-year	652/1,834,840	674/2,100,434	665/1,737,904	89/207,774		
Age- and sex- adjusted HR	1	0.88 (0.79, 0.98)	1.02 (0.92, 1.14)	1.20 (0.96, 1.50)	0.08	1.05 (0.99, 1.13)
Fully adjusted HR^1^	1	0.92 (0.82, 1.04)	1.01 (0.88, 1.15)	0.91 (0.71, 1.19)	0.83	0.96 (0.89, 1.05)

^1^ Adjusted for age (years), sex (M, F), use or non-use of pipes or cigars, the number of cigarettes smoked per day, time of smoking cessation (<1 year, 1 to <5 years, 5 to <10 years, or ≥10 years before baseline), alcohol drinking (grams per day), ethnicity (non-Hispanic White, non-Hispanic Black, Hispanic, Asian/Pacific Islander/Native American), body mass index (BMI)(kg/m^2^), education (high school or less, post-high school training, college graduate, post-graduate education), physical activity (never, rarely, 1–3 times per month, 1–2 times per week, 3–4 times per week, ≥ 5 times per week), self-report history of diabetes (yes, no), total energy intake (kcal per day, quintile), red meat intake, white meat intake, whole grain intake, fruit intake, and vegetable intake (grams per day, quintile); HRs (95% CIs) were calculated by using a Cox regression model

Mutual adjustment for each type of potato preparation did not change the results meaningfully. Likewise, using potato intake in grams consumed per day, adjusted for energy, did not change the results meaningfully (HR _C4 vs C1_ = 0.98, 95%CI = 0.95, 1.00).

**Fig 1:**
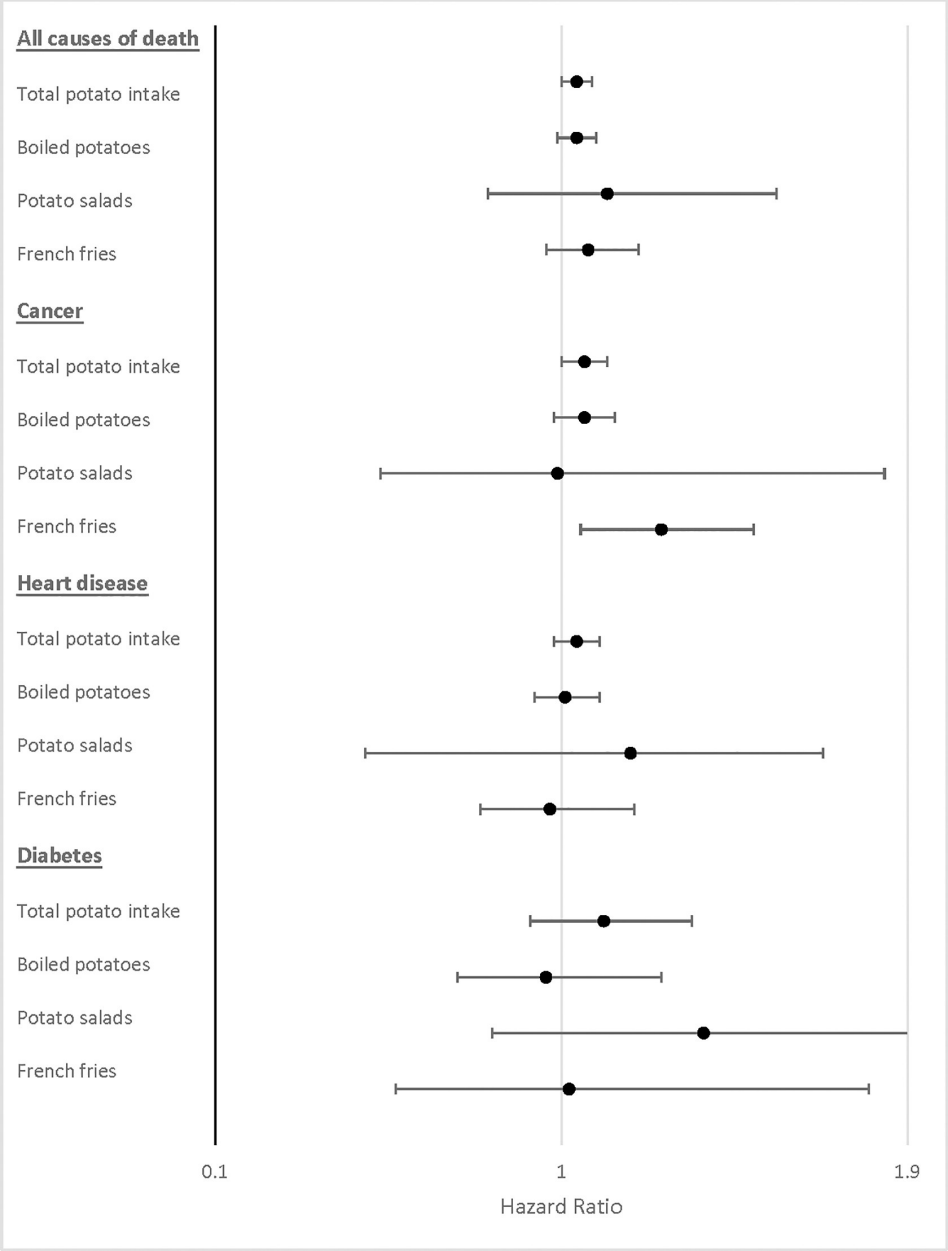
Hazard ratios for overall mortality for different types of potato preparation. Point estimates represent highest (≥7 times per week) vs. lowest category (<1 time per week) of consumption adjusted for age (years), sex (M, F), use or non-use of pipes or cigars, the number of cigarettes smoked per day, time of smoking cessation (<1 year, 1 to <5 years, 5 to <10 years, or ≥10 years before baseline), alcohol drinking (grams per day), ethnicity (non-Hispanic White, non-Hispanic Black, Hispanic, Asian/Pacific Islander/Native American), body mass index (BMI)(kg/m^2^), education (high school or less, post-high school training, college graduate, post-graduate education), physical activity (never, rarely, 1–3 times per month, 1–2 times per week, 3–4 times per week, ≥ 5 times per week), self-report history of diabetes (yes, no), calories (kcal per day, quintile), red meat intake, white meat intake, whole grain intake, fruit intake, and vegetable intake (grams per day, quintile); HRs (95% CIs) were calculated by using a Cox regression model.

We also investigated associations of several different types of potato preparations with mortality. Increasing consumption of “baked, boiled or mashed potatoes” or “potato salad” was not significantly associated with overall or cause specific mortality. However, French fry consumption was directly associated with cancer related mortality (HR _C4 vs C1_ = 1.27, 95%CI = 1.02, 1.59) (**Fig 1**).

**Fig 2:**
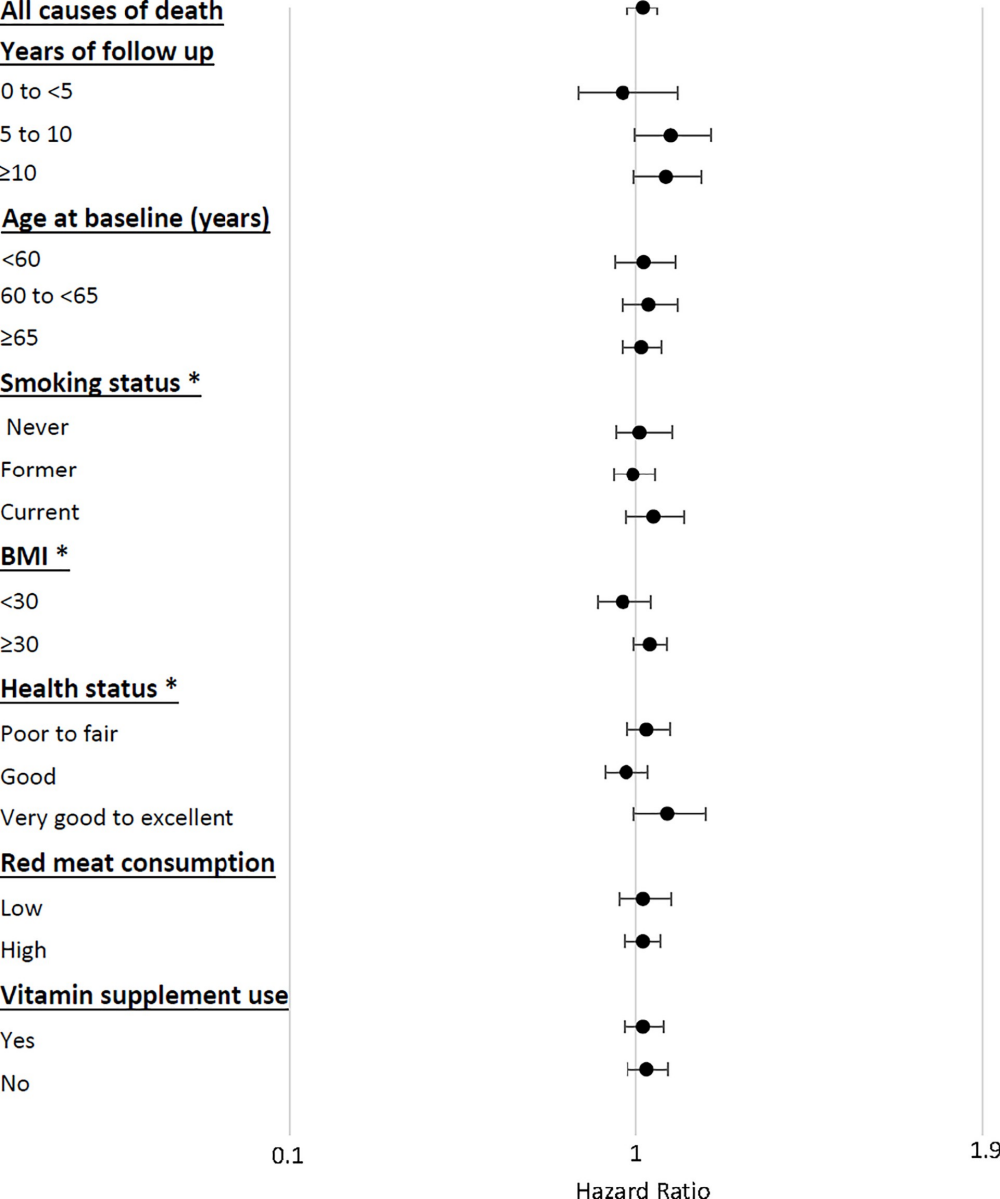
Hazard ratios for overall mortality, for total potato consumption stratified by categories of suspected risk factors. Point estimates represent highest vs. lowest category of potato consumption adjusted for age (years), sex (M, F),use or non-use of pipes or cigars, the number of cigarettes smoked per day, time of smoking cessation (<1 year, 1 to <5 years, 5 to <10 years, or ≥10 years before baseline), alcohol drinking (grams per day), ethnicity (non-Hispanic White, non-Hispanic Black, Hispanic, Asian/Pacific Islander/Native American), body mass index (BMI)(kg/m^2^), education (high school or less, post-high school training, college graduate, post-graduate education), physical activity (never, rarely, 1–3 times per month, 1–2 times per week, 3–4 times per week, ≥ 5 times per week), self-report history of diabetes (yes, no), total energy intake (kcal per day, quintile), red meat intake, white meat intake, whole grain intake, fruit intake, and vegetable intake (grams per day, quintile); HRs (95% CIs) were calculated by using a Cox regression model.* P for interaction <0.05.

We also performed stratified analyses according to categories of other risk factors for mortality. There were no substantial differences in the estimated associations across the strata of the various risk factors. The results of these stratified analyses are shown in **Fig 2**; no statistically significant associations were observed within any of the strata.

In a lag analysis, we excluded participants with less than two years of follow-up, and the results did not change meaningfully (HR _C4 vs C1_ = 1.04, 95%CI = 0.99, 1.09).

## Discussion

Our results do not support an association of potato intake with all-cause or cause-specific mortality. Potatoes have a high glycemic index and a high glycemic load. Consumption of high glycemic load foods may increase the risk of diabetes and cardiovascular diseases, independent of body weight change [14]. However, they are a good source of potassium, vitamin C, and polyphenols [3], and this may counterbalance the association of other potato components with mortality. Prospective studies of potato consumption and cardiovascular diseases have yielded inconsistent results [3, 15, 16]. However, our results are consistent with a systematic review study reported that potato consumption was not associated with the risk of obesity, diabetes or cardiovascular diseases [17]. A recent meta-analysis reported that potato consumption was not associated with risk of overall, cardiovascular, or cancer mortality [18]. Although there was no evidence for associations of potato intakes alone with mortality, we did not investigate potato consumption as a component of overall dietary glycemic load [19–21].

The different types of preparation methods could have different associations with health outcomes [22–24]. Baked potatoes have higher resistant starch contents than boiled potatoes [24]. A previous study found that the glycemic index of potatoes varies among different preparation method, but not among different types of potatoes tested [23]. We investigated associations of different types of potato preparations with mortality. Increasing consumption of “baked, boiled or mashed potatoes” or “potato salad” was not significantly associated with overall or cause specific mortality. We did not observe an association between French fry intake and total mortality. This result differs from a recent study which found that consumption of French fries 2–3 times per week was associated with higher risk of total mortality (7), but this previous study evaluated only 236 deaths during 8 years of follow-up [25]. In contrast, with nearly 77,000 deaths occurring during 15.6 years of follow-up, our study had much greater power to carefully evaluate the associations of multiple potato exposures with both total and cause-specific mortalities, and the only significant result of French fry consumption that we found was a weak direct association with cancer-related mortality. Fried potatoes contain acrylamides, which is a potential carcinogen [26, 27] but frequent french fry consumption may also be associated with other unhealthy lifestyle and poor dietary habits, which could lead to an apparent association in the presence of uncontrolled confounding. Given the multiple outcomes that we evaluated, even this result should be interpreted with caution, and we cannot rule out the possibility that this finding was the result of chance.

The strengths of this study were the large sample size, the long follow-up time, and the ability to adjust for many potential confounders. The most important methodological limitation of this study was the use of a single food frequency questionnaire to evaluate the diet. Food frequency questionnaires are known to be subject to measurement error, and the use of this instrument could be one of the factors contributing to the null findings of the current study. Also, recording dietary data only one time, at baseline, did not allow us to include changes in diet during the long follow-up time in our evaluation. In addition, although we adjusted the models for other potential confounders, residual confounding cannot be ruled out. We also lacked data on the amount of salt or other condiments added during the serving of foods, which might have affected our results.

In conclusion, we found that total potato consumption was not associated with risk of total mortality. Future studies in other populations are warranted given the high level of potato consumption and the equivocal results across the literature.

**Conflicts of interest:** None declared.

## Supporting information

S1 Fig Participant flow chart. (TIFF)

S1 Table Demographic and dietary characteristics of participants by different types of potato preparation in the NIH-AARP Diet and Health Study 1. (DOCX)

## Abbreviations

BMI: Body Mass Index
CI: Confidence Intervals
HR: Hazard Ratios
ICD-10: International Classification of Diseases, 10th revision
NIH-AARP: National Institutes of Health–American Association of Retired Persons Diet and Health Study
RDA: Recommended Dietary Allowance
