# Supplementary figures and images for: Potato consumption and the risk of overall and cause specific mortality in the NIH-AARP study
Source: PLoS One. 2019 May 7;14(5):e0216348. doi: 10.1371/journal.pone.0216348 (PMC6504095; doi:10.1371/journal.pone.0216348)

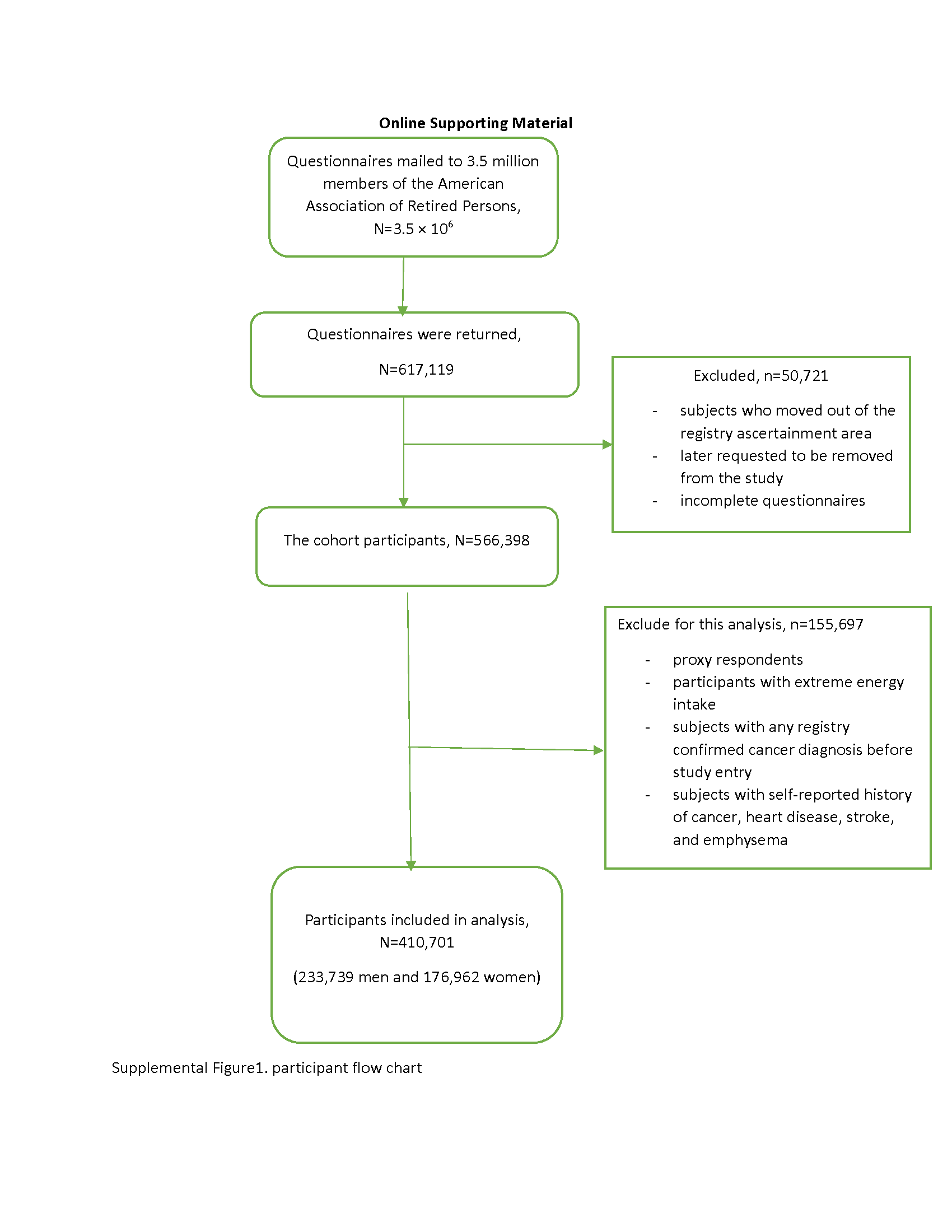

Supplement: S1 Fig — (TIFF) [file pone.0216348.s001.tiff]
