# Supplementary material for: Potato consumption and the risk of overall and cause specific mortality in the NIH-AARP study
Source: PLoS One. 2019 May 7;14(5):e0216348. doi: 10.1371/journal.pone.0216348 (PMC6504095; doi:10.1371/journal.pone.0216348)
Supplement: S1 Table — (DOCX) [file pone.0216348.s002.docx]

**Supplemental Table 1. Demographic and dietary characteristics of participants by different types of potato preparation in the NIH-AARP Diet and Health Study ^1^**

|  | **Boiled potatoes** | | | | **Potato salads** | | | | **French fries** | | | |
| --- | --- | --- | --- | --- | --- | --- | --- | --- | --- | --- | --- | --- |
|  | **< 1 time per week** | **1-2 times per week** | **3-6 times per week** | **≥7 times per week** | **< 1 time per week** | **1-2 times per week** | **3-6 times per week** | **≥7 times per week** | **< 1 time per week** | **1-2 times per week** | **3-6 times per week** | **≥7 times per week** |
| Participants, n | 159,523 | 139,597 | 105,129 | 6,452 | 400,165 | 9,468 | 969 | 99 | 317,503 | 70,842 | 21,381 | 975 |
| Potato intake, gram per day ^2^ | 0.05±0.02 | 0.2±0.0 | 0.5±0.1 | 1.03±0.2 | 0.2±0.2 | 0.3±0.2 | 0.40±0.3 | 0.4±0.4 | 0.2±0.2 | 0.3±0.2 | 0.3±0.2 | 0.4±0.4 |
| Sex, male (%) | 55.7 | 58.3 | 57.1 | 55.8 | 56.5 | 71.1 | 71.7 | 65.7 | 52.2 | 72.1 | 76.2 | 74.0 |
| Age, years ^2^ | 61.4±5.4 | 61.5±5.4 | 62.2±5.3 | 62.9±5.2 | 61.7±5.4 | 61.6±5.4 | 62.0±5.2 | 63.0±5.1 | 61.9±5.3 | 61.0±5.4 | 60.4±5.4 | 60.6±5.6 |
| BMI ^2^, kg/m^2^ | 27.0±5.1 | 27.0±4.9 | 27.0±5.0 | 26.8±5.6 | 27.0±5.0 | 27.4±5.0 | 27.5±5.5 | 26.1±5.0 | 26.8±5.0 | 27.7±4.9 | 28.3±5.3 | 28.4±5.8 |
| Smoking (%) |  |  |  |  |  |  |  |  |  |  |  |  |
| Never | 38.3 | 39.3 | 39.2 | 40.7 | 38.8 | 41.1 | 40.8 | 37.8 | 39.6 | 36.8 | 35.5 | 36.1 |
| Former <20 cigarettes per day | 29.5 | 28.9 | 28.4 | 27.9 | 29.0 | 27.1 | 24.6 | 26.7 | 29.6 | 27.1 | 25.0 | 24.6 |
| Former ≥20 cigarettes per day | 19.4 | 20.0 | 20.0 | 19.3 | 19.8 | 18.9 | 21.1 | 20.0 | 19.0 | 22.2 | 23.3 | 21.0 |
| Current <20 cigarettes per day | 8.8 | 7.7 | 7.8 | 8.2 | 8.2 | 8.1 | 9.7 | 13.3 | 8.0 | 8.4 | 9.1 | 9.9 |
| Current ≥20 cigarettes per day | 4.1 | 4.1 | 4.6 | 4.0 | 4.2 | 4.9 | 3.8 | 2.2 | 3.8 | 5.5 | 7.1 | 8.4 |
| Alcohol, grams per day ^2^ | 11.5±32.1 | 12.4±30.4 | 13.2±31.9 | 13.2±35.1 | 12.2±31.5 | 12.8±32.1 | 14.4±43.2 | 10.3±21.3 | 11.6±30.3 | 14.5±34.9 | 14.6±36.6 | 12.2±31.9 |
| Race (%) |  |  |  |  |  |  |  |  |  |  |  |  |
| Non-Hispanic white | 86.3 | 94.9 | 97.1 | 97.0 | 92.2 | 90.1 | 87.6 | 72.3 | 91.8 | 93.8 | 92.5 | 90.5 |
| Non-Hispanic Black | 7.1 | 2.7 | 1.6 | 1.5 | 4.0 | 6.1 | 7.5 | 19.2 | 4.4 | 3.0 | 3.7 | 4.4 |
| Others | 6.7 | 2.3 | 1.4 | 1.5 | 3.7 | 3.9 | 4.9 | 8.5 | 3.8 | 3.3 | 3.8 | 5.1 |
| Education, College and post-graduate (%) | 41.1 | 42.7 | 37.7 | 32.2 | 40.7 | 40.0 | 40.3 | 33.3 | 41.5 | 38.9 | 34.9 | 27.6 |
| Physical activity, ≥ 5 times per week (%) | 18.7 | 19.1 | 20.22 | 23.2 | 19.3 | 19.8 | 22.1 | 14.0 | 20.0 | 17.0 | 16.9 | 19.3 |
| Self-reported history of diabetes, yes (%) | 7.5 | 6.9 | 7.4 | 8.9 | 7.2 | 9.4 | 10.7 | 12.1 | 6.9 | 8.1 | 9.7 | 12.6 |
| Self-reported poor or fair health (%) | 8.3 | 7.1 | 7.5 | 9.8 | 7.6 | 10.2 | 11.6 | 11.2 | 7.4 | 8.2 | 10.0 | 13.4 |
| Use of any vitamin mineral supplement (%) | 56.3 | 56.2 | 54.9 | 51.7 | 55.9 | 54.6 | 53.0 | 49.5 | 57.2 | 51.7 | 49.1 | 47.3 |
| Calories, kcal/day ^2^ | 1588±739 | 1863±751 | 2121±827 | 2460±960 | 1813±788 | 2530±978 | 2745±1137 | 2712±1186 | 1709±731 | 2148±838 | 2535±980 | 2967±1108 |
| Red meat intake, grams per day ^2^ | 52.4±48.9 | 69.0±54.8 | 79.9±62.3 | 87.1±72.6 | 64.6±55.1 | 107.5±77.0 | 107.3±89.5 | 80.3±75.4 | 55.3±47.2 | 94.3±62.5 | 120.6±81.2 | 139.8±100.6 |
| White meat intake, grams per day ^2^ | 50.8±49.5 | 61.8±49.7 | 67.8±55.8 | 74.0±71.9 | 58.6±51.5 | 83.9±66.6 | 90.4±82.2 | 82.6±75.2 | 57.5±51.4 | 64.0±51.8 | 69.9±60.8 | 75.0±72.9 |
| Whole grain intake, grams per day ^2^ | 19.2±27.1 | 22.7±28.2 | 26.1±31.7 | 33.3±41.7 | 22.3±29.1 | 26.0±32.8 | 28.0±36.3 | 32.1±42.9 | 22.3±28.8 | 22.4±29.5 | 23.8±32.3 | 28.0±39.3 |
| Fruit intake, grams per day ^2^ | 348.5±341.8 | 367.5±318.8 | 391.4±336.6 | 463.6±422.9 | 365.7±332.8 | 438.7±388.7 | 484.8±469.5 | 537.0±497.8 | 374.1±337.6 | 346.0±317.6 | 344.9±340.6 | 385.3±436.6 |
| Vegetable intake, grams per day ^2,3^ | 212.6±177.9 | 257.1±173.7 | 298.0±203.0 | 373.6±293.7 | 249.6±187.3 | 340.1±226.2 | 388.4±305.1 | 354.0±33.4 | 250.1±191.3 | 251.8±173.0 | 277.9±200.2 | 341.8±306.0 |

^1^ All risk factors were associated with potato consumption with p <0.001

^2^ Mean ±SD

^3^ Excluding potatoes
